# Early identification of intensive care unit-acquired infections with daily monitoring of C-reactive protein: a prospective observational study

**DOI:** 10.1186/cc4892

**Published:** 2006-04-24

**Authors:** Pedro Póvoa, Luís Coelho, Eduardo Almeida, Antero Fernandes, Rui Mealha, Pedro Moreira, Henrique Sabino

**Affiliations:** 1 Unidade de Cuidados Intensivos, Hospital Garcia de Orta, Almada, Portugal

## Abstract

**Introduction:**

Manifestations of sepsis are sensitive but are poorly specific of infection. Our aim was to assess the value of daily measurements of C-reactive protein (CRP), temperature and white cell count (WCC) in the early identification of intensive care unit (ICU)-acquired infections.

**Methods:**

We undertook a prospective observational cohort study (14 month). All patients admitted for ≥72 hours (*n *= 181) were divided into an infected (*n *= 35) and a noninfected group (*n *= 28). Infected patients had a documented ICU-acquired infection and were not receiving antibiotics for at least 5 days before diagnosis. Noninfected patients never received antibiotics and were discharged alive. The progression of CRP, temperature and WCC from day -5 to day 0 (day of infection diagnosis or of ICU discharge) was analyzed. Patients were divided into four patterns of CRP course according to a cutoff value for infection diagnosis of 8.7 mg/dl: pattern A, day 0 CRP >8.7 mg/dl and, in the previous days, at least once below the cutoff; pattern B, CRP always >8.7 mg/dl; pattern C, day 0 CRP ≤8.7 mg/dl and, in the previous days, at least once above the cutoff; and pattern D, CRP always ≤8.7 mg/dl.

**Results:**

CRP and the temperature time-course showed a significant increase in infected patients, whereas in noninfected it remained almost unchanged (*P *< 0.001 and *P *< 0.001, respectively). The area under the curve for the maximum daily CRP variation in infection prediction was 0.86 (95% confidence interval: 0.752–0.933). A maximum daily CRP variation >4.1 mg/dl was a good marker of infection prediction (sensitivity 92.1%, specificity 71.4%), and in combination with a CRP concentration >8.7 mg/dl the discriminative power increased even further (sensitivity 92.1%, specificity 82.1%). Infection was diagnosed in 92% and 90% of patients with patterns A and B, respectively, and in only two patients with patterns C and D (*P *< 0.001).

**Conclusion:**

Daily CRP monitoring and the recognition of the CRP pattern could be useful in the prediction of ICU-acquired infections. Patients presenting maximum daily CRP variation >4.1 mg/dl plus a CRP level >8.7 mg/dl had an 88% risk of infection.

## Introduction

Nosocomial infections are an increasingly common cause of morbidity and mortality [1], particularly among critically ill patients [2,3]. In intensive care units (ICUs), clinicians are repeatedly faced with two challenges: whether a patient is infected and whether antibiotic therapy is doing any good. Sepsis is defined as the host response to an infection and is characterized by a number of signs such as fever, tachycardia, tachypnea and leukocytosis [4,5]. These signs are very sensitive but are poorly specific of infection, can occur in a variety of noninfectious conditions [6,7] and can be influenced by commonly used drugs [8]. Untreated bacterial infections may cause serious complications, but treating noninfectious causes with antimicrobials is ineffective and also increases costs, toxicity and the risk of development of bacterial resistance. A better knowledge of the inflammatory cascade has given new insights and provided several mediators that [9], in conjunction with the clinical manifestations of sepsis, can be useful as markers of infection. C-reactive protein (CRP) is one such mediator and is probably the most widely used marker [10-12].

CRP is an acute-phase protein, stably conserved throughout vertebrate evolution, suggesting a central role in immunological response [13]. It is synthesized in the liver mainly in response to IL-6 and binds to polysaccharides of pathogens promoting phagocytosis [14]. Several studies have shown that CRP could be useful in infection diagnosis [10] as well as in monitoring the response to antibiotic therapy [12,15].

As CRP measurement is a rapid, reproducible and inexpensive test, the aim of our study was to evaluate whether daily CRP monitoring as well as the assessment of CRP patterns of progression could be useful in the early identification of patients with ICU-acquired infections, in comparison with commonly used markers such as temperature and white cell count (WCC).

## Materials and methods

The study was conducted in an eight-bed medico-surgical ICU of the Garcia de Orta Hospital, Almada, Portugal, which admits patients from all hospital departments as well as from other hospitals. Between November 2001 and December 2002 all patients admitted to the ICU who were ≥18 years old and stayed 72 hours or longer were potentially eligible. For patients with multiple ICU admissions, only the first admission was recorded. The Ethics Committee of Garcia de Orta Hospital approved the study design and informed consent was waived in view of the lack of need for additional blood sampling.

Data collected included the admission diagnosis, past medical history, vital signs, systemic inflammatory response syndrome (SIRS) [4], the Acute Physiology and Chronic Health Evaluation II (APACHE II) score [16] and the Sequential Organ Failure Assessment (SOFA) score [17]. CRP and WCC were measured at admission and then daily until discharge or death. The temperature was evaluated hourly and daily extreme values were recorded. Patients were evaluated daily for clinical evidence of infection, and samples for bacteriological cultures were collected whenever clinical suspicion was present.

A prospective cohort study design was used segregating only infected patients and noninfected patients. Infected patients were those with an ICU-acquired infection according to the Centers for Disease Control definitions [18], those with positive cultures and those who were not receiving antibiotics for at least 5 days before infection diagnosis. Noninfected patients had no bacteriological or clinical signs of infection, had never received antibiotics and were discharged alive from the ICU. For purposes of the time-dependent analysis, day 0 was defined as the day of positive cultures in infected patients and as the day of ICU discharge in noninfected patients.

Blood samples were obtained from an arterial line at admission and subsequently every morning at 07:00. Measurement of CRP was made by an immunoturbidimetric method using a commercially available kit (Tina-quant CRP; Roche Diagnostics, Mannheim, Germany). The precision of the assay calculated by the intra-assay and inter-assay coefficient of variation was <7%, the sensitivity of the method was 0.1 mg/dl and the detection limit was 0.3 mg/dl.

Some additional variables were analyzed: the maximum daily CRP, temperature and WCC variations (calculated by computing the greatest absolute difference from the previous day's level) and the ΔCRP (calculated by computing day 0 concentrations minus the lowest CRP value).

**Figure 1:**
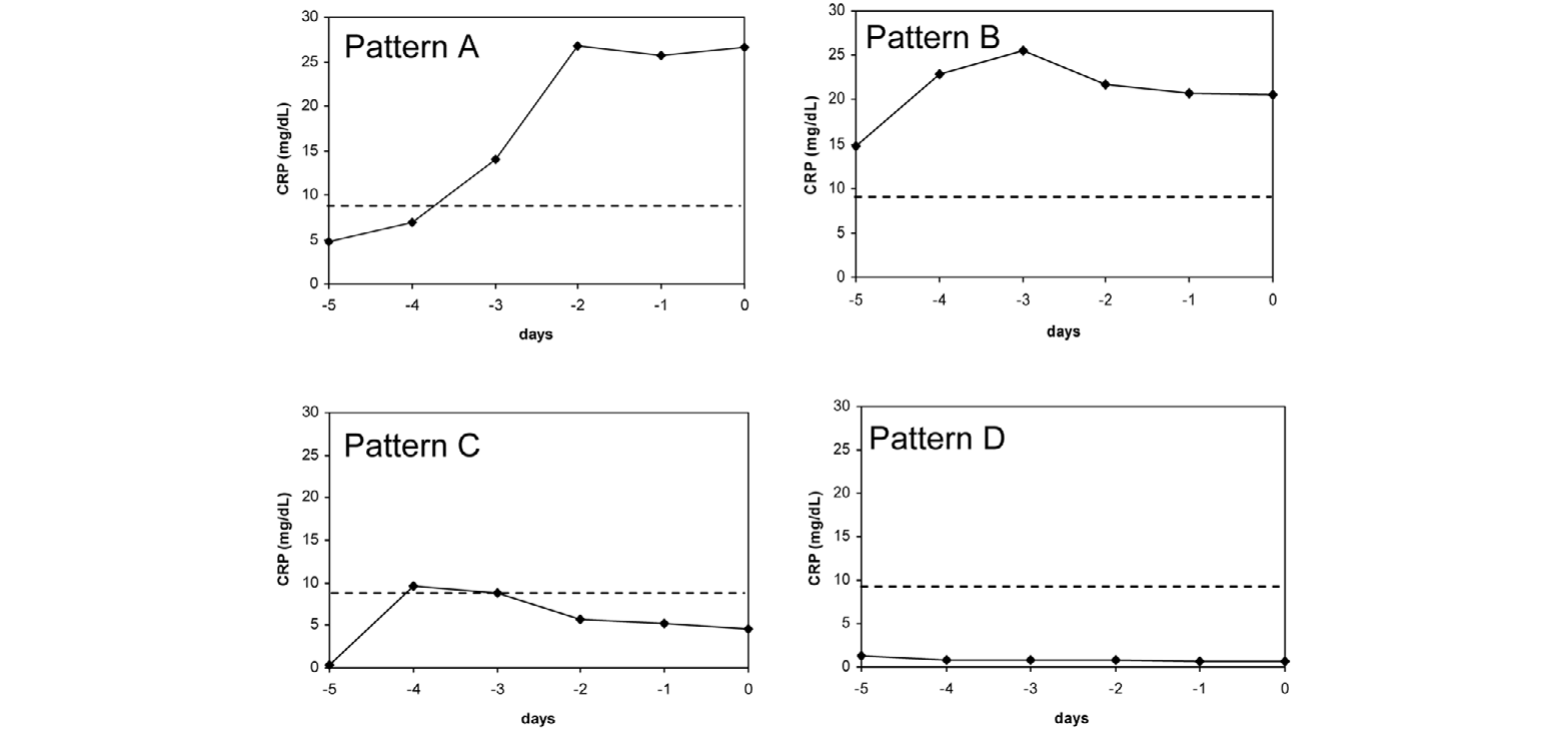
Patterns of C-reactive protein (CRP) course before infection diagnosis or intensive care unit discharge. Four patterns of CRP course between day -5 and day 0 before infection diagnosis or intensive care unit discharge of individual patients are displayed according to a previously defined CRP cutoff value for infection diagnosis of 8.7 mg/dl [19]. See text for definition of patterns A–D. Dashed line, CRP cutoff value for infection diagnosis.

We defined four patterns of CRP course before infection diagnosis or discharge (Figure 1) according to a previously identified CRP cutoff value for infection diagnosis of 8.7 mg/dl [19]. Pattern A occurred when the day 0 CRP was >8.7 mg/dl and, in the previous days, was at least once below the cutoff value. Pattern B occurred when CRP was always >8.7 mg/dl. Pattern C occurred when the day 0 CRP was ≤8.7 mg/dl and, in the previous days, was at least once above the cutoff value. Finally, pattern D occurred when CRP was always ≤8.7 mg/dl.

The progression of CRP, temperature, WCC and SOFA score from day -5 to day 0 was analyzed, comparing infected patients and noninfected patients. Patients were also retrospectively classified according to the individual CRP pattern, assessing its correlation with the clinical course.

### Statistical analysis

Results are expressed as the mean ± standard deviation unless stated otherwise. To assess differences between the two main groups the Student's *t *test and the Mann-Whitney *U* test were used for continuous variables and the χ^2 ^test was used for categorical variables. Time-dependent analysis of different variables was performed with general linear model, univariate, repeated-measures analysis using a split-plot design approach.

Receiver operating characteristics curves were plotted for the maximum daily CRP, temperature and WCC variations, and for ΔCRP. The accuracy of these variables was assessed by calculating the area under the curve (AUC). In medical practice, a diagnostic test with an AUC <0.75 would be regarded as noncontributive [20].

We created a multivariable logistic regression model to determine independently associated risk factors best predicting infection. The studied variables as infection predictors, specifically the maximum daily CRP, temperature and WCC variations, and ΔCRP as well as the age, sex, APACHE II score and admission diagnoses, were considered for the multivariable logistic regression model if they were statistically significant in bivariate analyses (*P *< 0.05) and if they had an odds ratio ≥1.2. Before entering the logistic regression model, multicolinearity among risk factors was checked by computing the correlation coefficient (*r*) between variables taken two by two; *r *< 0.4 was considered low enough to exclude correlation between the risk factors. Model calibration and discrimination were assessed using the Hosmer-Lemeshow goodness-of-fit test and the *c *statistic, respectively. Results were reported as the odds ratio with the 95% confidence interval. Significance was accepted for *P *< 0.05. Statistical analyses were performed with the use of SPSS software (version 10.0; SPSS Inc., Chicago, Illinois, USA).

## Results

There were 260 patients admitted to our ICU during the study period, with 181 (69.6%) staying for 72 hours or longer. Of these patients, 32 never received antibiotics during the ICU stay. Twenty-eight (15.5%) out of these 32 patients without antibiotics were discharged alive from the ICU, making up the noninfected group. The occurrence of documented ICU-acquired infections in patients not receiving antibiotics for at least 5 days was diagnosed in 19.3% (*n *= 35), constituting the infected group (Table 1). The remaining 114 patients were excluded from the final analysis.

The number of days without antibiotics before infection diagnosis in infected patients and the length of stay among noninfected patients were 6.7 ± 2.9 days and 5.7 ± 3.5 days, respectively (*P *= 0.055). Infection was mostly due to bacteria (97%), and more than one pathogen was isolated in two cases.

The median (interquartile range) CRP concentrations in infected and noninfected patients at day 0 were 16.6 (9.1) mg/dl and 3 (4.5) mg/dl, respectively (*P *< 0.001). The temperature in infected patients was also significantly higher than in the noninfected group (38.1 ± 1.0°C and 37.1 ± 0.6°C, respectively; *P *< 0.001). The WCC values were equally elevated in both groups (15 ± 8.6 × 10^3^/mm^3 ^and 11.7 ± 4 × 10^3^/mm^3^, respectively; *P *= 0.496).

**Figure 2:**
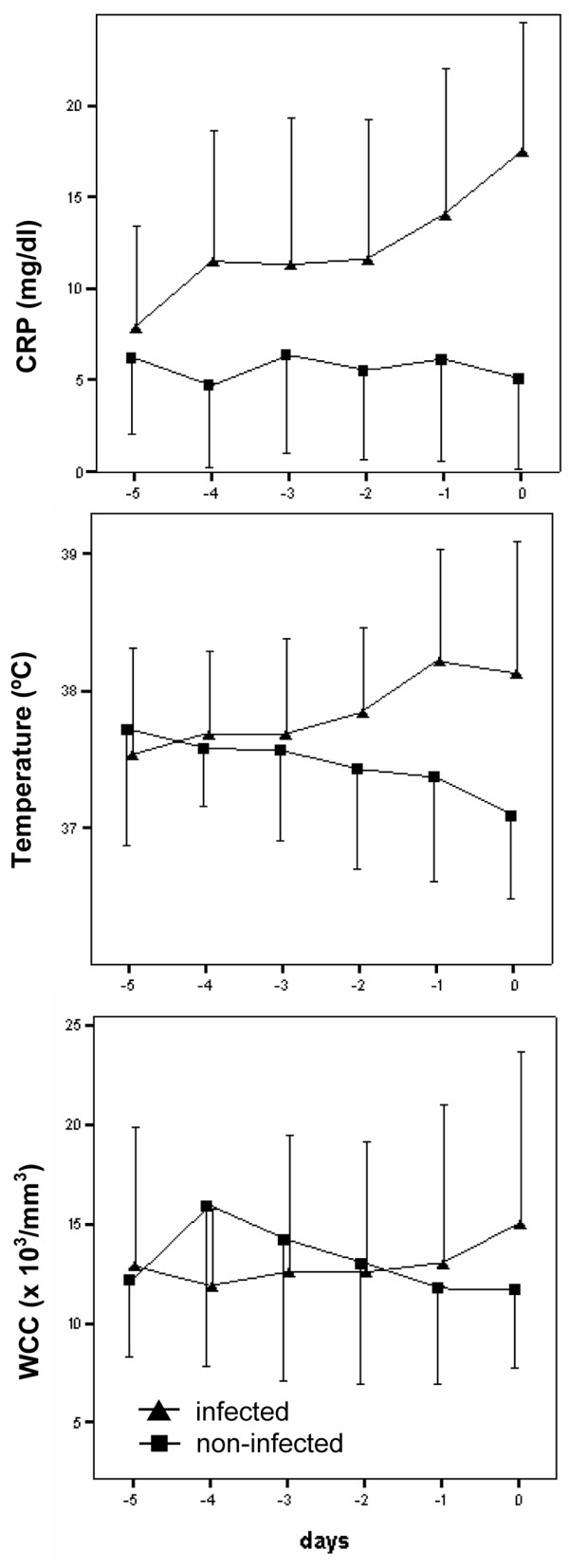
C-reactive protein (CRP), temperature and white cell count (WCC) progression before infection diagnosis or discharge. The time-dependent analysis of CRP, temperature and WCC (mean ± standard deviation) from day -5 to day 0 of infected patients and noninfected patients is presented. Both the CRP and temperature course clearly differentiate infected patients from noninfected patients (*P *< 0.001 and *P *< 0.001, respectively). Although the WCC time-dependent analysis was significantly different (*P *= 0.005), its progression was unpredictable and erratic both in infected patients as well as in noninfected patients.

Time-dependent analysis of CRP (Figure 2) during the 5 days before the event of interest showed a steady and significant increase in infected patients, more than twofold, whereas the CRP level in noninfected patients remained almost unchanged (*P *< 0.001). Over the same period, the temperature increased significantly in infected patients while it decreased slightly in noninfected patients (*P *< 0.001) (Figure 2). The time-dependent analysis of WCC showed a significant difference between infected and noninfected patients (*P *= 0.005), but this finding resulted from an unpredictable and erratic progression (Figure 2). As a result, WCC comparisons of infected and noninfected patients between day -5 and day 0 were not significantly different: from 12.9 ± 6.9 to 15 ± 8.6 × 10^3^/mm^3 ^(*P *= 0.168) and from 12.2 ± 3.9 to 11.7 ± 4 × 10^3^/mm^3^, respectively (*P *= 0.779).

We then analyzed the maximum daily CRP, temperature and WCC variations during the study period. The AUC of the maximum daily CRP variation as a predictor of infection was 0.86 (95% confidence interval: 0.752–0.933). An increase in CRP >4.1 mg/dl was a marker of infection prediction with a sensitivity of 92.1% and a specificity of 71.4% (positive likelihood ratio 3.22, negative likelihood ratio 0.11). The AUCs of the maximum daily temperature and WCC variations as a predictor of infection were both <0.75: 0.739 (95% confidence interval: 0.616–0.839) and 0.668 (95% confidence interval: 0.541–0.779), respectively. Finally, we also plotted the receiver operating characteristics curve of ΔCRP with an area of 0.879 (95% confidence interval: 0.775–0.946). ΔCRP >5 mg/dl was a marker of infection prediction with a sensitivity of 81.6% and a specificity of 89.3% (positive likelihood ratio 7.61, negative likelihood ratio 0.21).

Among the eight variables (maximum daily CRP, temperature and WCC variations, ΔCRP, age, sex, APACHE II and admission diagnoses) entered as independent variables in the bivariate logistic regression equation, only four (maximum daily CRP, temperature and WCC variations, and ΔCRP) were found to be good predictors of infection (*P *< 0.05 and odds ratio ≥1.2). A significant colinearity was found between the maximum daily CRP variation and ΔCRP (*r *= 0.507). As a result ΔCRP was not entered in the final model. The multivariable logistic regression analysis (Table 2) found that only the maximum daily CRP variation was an independent predictor of infection (model *n *= 63, 35 of which developed infection; AUC = 0.899, goodness-of-fit = 0.593).

Furthermore, we assessed the discrimination between infected and noninfected patients according to the cutoff value for infection diagnosis of CRP (>8.7 mg/dl) and temperature (>38.2°C) published elsewhere [19]. In only one infected patient were all CRP values below the cutoff value during the study period, while eight noninfected patients presented CRP >8.7 mg/dl at least once (*P *< 0.001). Similarly, concerning temperature >38.2°C, 28 infected patients and 10 noninfected patients showed such a temperature at least once (*P *= 0.002). Among the 35 infected patients, 26 showed both a maximum daily CRP variation >4.1 mg/dl and a temperature >38.2°C. These variations took place simultaneously in seven patients. A temperature above the cutoff value occurred before the CRP variation in seven patients, whereas in 12 patients the CRP changed first.

In the study period, the combination of a maximum daily CRP variation >4.1 mg/dl plus a concentration >8.7 mg/dl further increased the discriminative power for infection diagnosis with a sensitivity of 92.1% and a specificity of 82.1% (positive likelihood ratio 5.2, negative likelihood ratio 0.1).

### Patterns of the CRP course before infection diagnosis

Patients were retrospectively divided according to the pattern of CRP evolution during the 5 days before the event of interest (Figure 1). Twenty-six patients were classified as pattern A, 10 patients as pattern B, six patients as pattern C and 21 patients as pattern D. The time-dependent analysis of the different CRP patterns showed that these patterns of evolution were statistically different (*P *< 0.001). Almost all patients with patterns A and B (92% and 90%, respectively) developed an ICU-acquired infection. On the contrary, only one patient classified as pattern C and one patient classified as pattern D became infected (*P *< 0.001). No relationship between the source of infection and the CRP pattern of evolution was found (*P *= 0.748). Time-dependent analysis of temperature according to the predefined CRP patterns was also significantly different (*P *< 0.001). Together patients with patterns A and B showed an increase in temperature, although not reaching significance (*P *= 0.363), whereas a significant decrease was observed in those patients with patterns C and D (*P *= 0.001).

### Correlation between clinical course and infection diagnosis

Clinical evolution during the study period was monitored with daily assessment of SIRS and the SOFA score. SIRS was present in 95% of infected patients at day 0 as well as in 82% of the patients ready to be discharged (*P *= 0.101). The same was true in the days before the event of interest.

**Figure 3:**
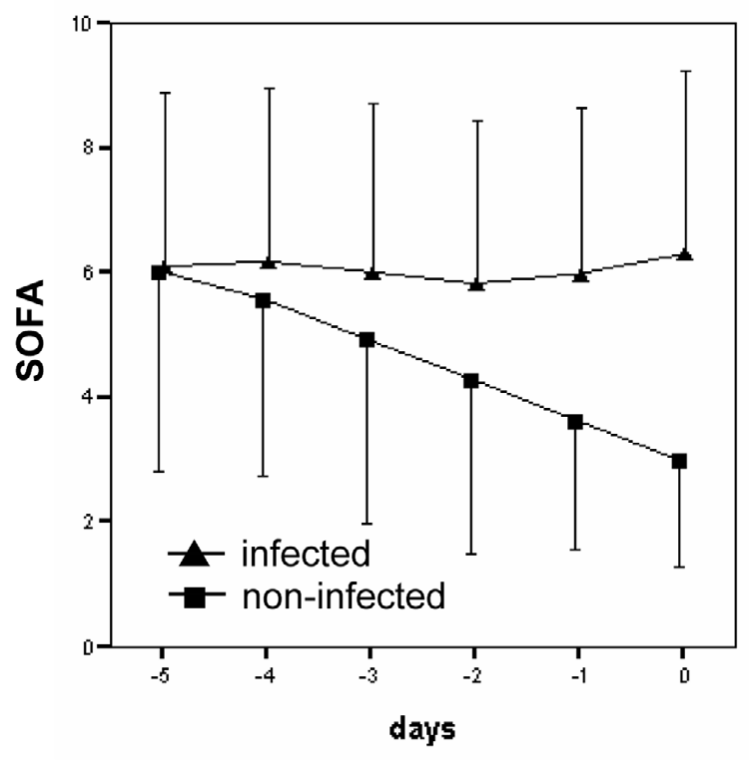
Clinical course evaluated by the Sequential Organ Failure Assessment (SOFA) score in infected and noninfected patients. The SOFA score (mean ± standard deviation) between day -5 and day 0 of infected patients and noninfected patients is shown. In infected patients the SOFA score remained almost unchanged, whereas a significant decrease was observed in noninfected patients (*P *< 0.001).

The SOFA score (Figure 3) was significantly different between both groups (*P *< 0.001). In infected patients the SOFA score remained almost unchanged from day -5 to day 0 (6.0 ± 3.2 and 6.3 ± 2.9, respectively; *P *= 0.332), whereas in noninfected patients a significant decrease was observed (from 6.1 ± 2.8 to 3.0 ± 1.7, *P *= 0.011).

Finally, time-dependent analysis of the SOFA score of the four CRP patterns showed that the patterns of evolution were significantly different (*P *= 0.002). SOFA scores at day -5 of patients with patterns A, B, C and D were 5.9 ± 3.1, 6.8 ± 1.9, 6.0 ± 1.0 and 5.7 ± 3.9, respectively (*P *= 0.91, with one-way analysis of variance). Later on, at day 0, the SOFA score changed to 6.0 ± 3.1, 6.6 ± 2.8, 3.3 ± 1.6 and 3.0 ± 1.9, respectively (*P *< 0.001, with one-way analysis of variance).

## Discussion

Numerous studies have evaluated the usefulness of different markers, such as CRP [10,19,21] and procalcitonin [10,22], both in the diagnosis of and in the identification of patients at risk of infection. These concepts deserve further clarification. A marker of infection is not present before infection, it appears concomitantly and ideally precedes the infection, and it disappears with successful therapy or remains elevated if infection is refractory to treatment [23]. A risk factor of infection is a sign that identifies a group of patients at risk of developing an infection in the future.

The majority of published studies [10,11,21,24] evaluated the discriminative power for infection diagnosis of a single determination of a particular marker. These variables are not static, however, but dynamic, as their concentration depends on the intensity of the inflammatory stimulus; in particular, bacterial infection. As a result, the aim of the present study was to evaluate whether serial CRP measurements could be useful as an early predictor of infection.

Both fever and leukocytosis are classic markers of infection. Body temperature has a poor diagnostic performance for infection. A substantial proportion of infected patients are not febrile [25], fever is frequently not caused by an infection [6,7] and temperature is influenced by several noninfectious factors, such as antipyretics. In our group of patients, fever (defined as a body temperature >38.2°C [19]) was associated with infection in almost three-quarters of the febrile patients.

An increase in the WCC is also typically associated with infection, although leukopenia can also occur [4,26]. The WCC is also influenced by several noninfectious factors, such as corticoids. As a result, several studies found that WCC had a low diagnostic performance for infection [10,11,19,27]. The same was true in our series.

Interestingly, several authors found that an infection should be suspected with a steady CRP increase over 2 or 3 days, in the absence of any intervention able to mount an inflammatory response, such as surgery [10,28-30]. To our knowledge, there is only one study that has looked at the behavior of CRP before infection diagnosis [31]. In that study, performed with critically ill patients, a 25% or greater increase in the CRP concentration from the previous day's level was highly suggestive of infection. Additionally, several reports with trauma and surgical patients have demonstrated that a failure of CRP levels to fall or a secondary rise of CRP levels was highly suggestive of an infectious complication [28,32-34]. Our results showed that a maximum daily CRP variation >4.1 mg/dl from the previous day's level was highly suggestive of an ICU-acquired infection, and if in addition the absolute CRP concentration reached 8.7 mg/dl [19], it further increased the predictive value for infection. In our series, infection developed in 88% of the patients with both criteria.

The presence of SIRS was never helpful in distinguishing infected patients from noninfected patients, as other studies have already pointed out [35,36]. Conversely, we found a significant and steady decrease of the SOFA score in noninfected patients while the SOFA score in infected patients remained elevated without significant changes. We went further in our analysis to assess the relationship between CRP patterns with the SOFA score. Patients with patterns A and B showed a persistently elevated organ failure, while the SOFA score decreased steadily over time in patients with patterns C and D.

Some limitations to our investigation should be noted. This was a cohort single-center observational study using variables that are collected daily and are readily available at the bedside with the aim of predicting infection. In addition, the study included only ICU-acquired infections.

Moreover, some strengths of our work should be addressed. Apart from the study of Matson and colleagues [31], we are not aware of any other report investigating the usefulness of serial measurements of a sepsis marker to predict infection in critically ill patients. In addition, we identified different patterns of CRP progression, with different clinical courses and correlations with infection. As a result, we speculate that infection should be strongly suspected in patients with patterns A and B, and consequently a thorough diagnostic work-up should be performed. In contrast, infection is considered very unlikely in patients with patterns C and D, and antibiotic therapy could eventually be withheld in the absence of a strong clinical suspicion of infection.

## Conclusion

The data of the present study indicate that daily CRP determinations could be useful as a marker of infection prediction, since patients presenting a maximum daily CRP variation >4.1 mg/dl plus a CRP level >8.7 mg/dl had an 88% risk of ICU-acquired infection. In addition, the recognition of the patterns of CRP progression adds more information about the individual clinical course. Both the temperature and WCC were not very useful as markers of infection prediction. Serial CRP measurements might consequently be of some help in the clinical decision-making process; namely, guiding culture sampling as well as empirical prescription of antibiotics. Further studies to assess the clinical impact of daily CRP monitoring should be performed.

## Key messages

• 	Daily CRP determinations could be useful as a marker of infection prediction because patients presenting a maximum daily CRP variation >4.1 mg/dl plus a CRP level >8.7 mg/dl had an 88% risk of ICU-acquired infection. Both the temperature and WCC were not very useful as markers of infection prediction.

• 	The presence or absence of SIRS criteria was never helpful in distinguishing infected patients from noninfected patients.

• 	Four CRP patterns could be identified in infected patients before infection diagnosis and in noninfected patients before ICU discharge, which showed diverse associations with prediction of infection. The recognition of the individual CRP pattern adds valuable information about a patient's clinical course.

• 	Serial CRP measurements might be of some help in the clinical decision-making process; namely, guiding culture sampling as well as the empirical prescription of antibiotics.

## Abbreviations

APACHE II = Acute Physiology and Chronic Health Evaluation II; AUC = area under the curve; CRP = C-reactive protein; ICU = intensive care unit; IL = interleukin; SIRS = systemic inflammatory response syndrome; SOFA = Sequential Organ Failure Assessment; WCC = white cell count.

## Competing interests

The authors declare that they have no competing interests.

## Authors' contributions

PP conceived the study. All authors participated in the original design and in writing the original protocol. PP and LC collected and analyzed the data and drafted the manuscript. EA, AF, RM, PM and HS helped with manuscript drafting. All authors read and approved the final manuscript.

## Figures and Tables

**Table 1:** Demographic characteristics of the infected and noninfected patients

General characteristic	Noninfected patients (*n *= 28)	Infected patients (*n *= 35)	*P*
Age (mean ± standard deviation)	50.6 ± 21.9	62.2 ± 13.3	0.05
Sex (male/female)	13/15	24/11	0.08
APACHE II score (mean ± standard deviation)	17.3 ± 9.3	20.5 ± 6.1	0.11
Primary admission intensive care unit diagnosis (*n*)			0.063
Respiratory	4	11	
Cardiovascular	8	7	
Neurology	6	3	
Surgical	1	5	
Trauma	3	7	
Obstetrics	4		
Others	2	2	
Primary sites of infection (*n*)			
Respiratory		20	
Blood		11	
Gastrointestinal		3	
Skin and soft tissues		1	
SOFA score, day 0 (mean ± standard deviation)	3.0 ± 1.7	6.3 ± 2.9	<0.001
C-reactive protein, day 0 [median (interquartile range)]	3.0 (4.5)	16.6 (9.1)	<0.001

APACHE II, Acute Physiology and Chronic Health Evaluation II score; SOFA, Sequential Organ Failure Assessment.

**Table 2:** Results of multivariable logistic regression model

	Odds ratio	95% confidence interval	*P*
Maximum daily C-reactive protein variation	1.508	1.201–1.892	<0.001
Maximum daily temperature variation	1.126	0.994–1.275	0.061
Maximum daily white cell count variation	1.090	0.857–1.388	0.483

Variations per unit of measurement (1 mg/dl C-reactive protein; 0.1°C temperature; 1 × 10^3^/mm^3 ^white cell count).
